# Anti-leukemia effects of ginsenoside monomer: A narrative review of pharmacodynamics study

**DOI:** 10.1016/j.curtheres.2024.100739

**Published:** 2024-02-25

**Authors:** Seyyed Mohammad Matin Alavi Dana, Mohammadreza Meghdadi, Saeed Khayat Kakhki, Reza Khademi

**Affiliations:** 1 Student Research Committee, Faculty of Medicine, Mashhad University of Medical Sciences, Mashhad, Iran; 2 Department of Hematology and Blood Banking, Faculty of Medical Science, Mashhad University of Medical Science, Mashhad, Iran; 3 Department of Gerontological Nursing, School of Nursing, Social Development and Health Promotion Research Center, Gonabad University of Medical Sciences, Gonabad, Iran

**Keywords:** Ginseng, Ginsenoside, Leukemia, Cancer

## Abstract

**Background:**

Leukemia is a prevalent disease with high mortality and morbidity rates. Current therapeutic approaches are expensive and have side effects.

**Objective:**

In this investigation, we reviewed studies that investigated the anticancer effects of ginsenoside derivatives against leukemia and also explained the three main Ginsenoside derivatives (ginsenoside Rg3, Rh2, and Rg1) separately.

**Methods:**

An extensive search was conducted in Pubmed, Web of Science, and Google Scholar and relevant studies that investigated anticancer effects of ginsenoside derivatives against leukemia cancer were extracted and reviewed.

**Results:**

Preclinical studies reported that ginsenoside derivatives can induce apoptosis, suppress the proliferation of cancer cells, and induce differentiation and cell cycle arrest in leukemia cells. in addition, it can suppress the chemokine activity and extramedullary infiltration of leukemia cells from bone marrow. using herbal medicine and its derivatives is a promising approach to current health problems.

**Conclusion:**

This review shows that ginsenoside derivatives can potentially suppress the growth of leukemia cells via various pathways and can be applied as a new natural medicine for future clinical research.

## Graphical Abstract

**Figure:**
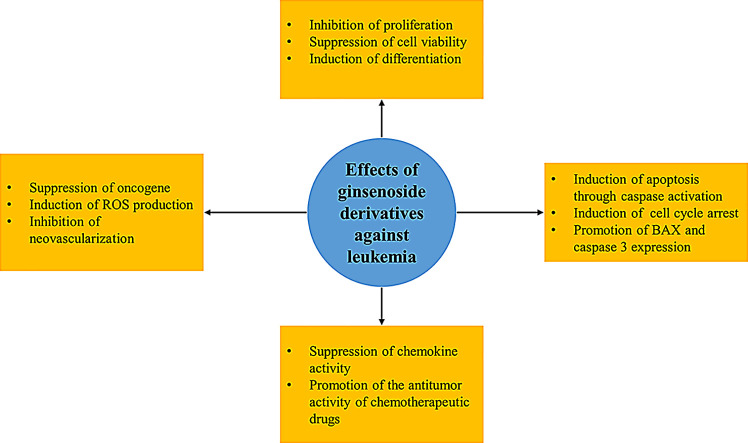

## Introduction

Leukemia is a hematologic malignancy that results from the uncontrolled proliferation of leukocytes and originates from myelocytes or lymphocytes. It causes different complications including organ infiltration and pancytopenia, resulting in bleeding due to thrombocytopenia, and increased risk of infection due to leukopenia.^1^ Unfortunately, due to the high prevalence and expensive treatments of leukemia, it has high economic burden. On the other hand, Patients with leukemia have poor prognosis and survival. Studies show that the 5-year survival rate of younger patients and one year in older patients is 50 percent.^2^ There are several challenges with the management and treatment of patients with leukemia. Currently approved drugs for leukemia have side effects and are expensive treatments for example, Bruton tyrosine kinase inhibitors can cause atrial fibrillation arrhythmia in the heart.^3^ Relapse of disease is another problem in the management of acute leukemia.^4^^,^^5^ Thus, investigating novel therapeutic natural products that are safe and efficient in the treatment of leukemia is very necessary.

Ginsenosides are derived from Panax ginseng and contain different. Many studies show that ginsenoside derivatives have anticancer effects against leukemia cancer cells. For instance, in a study by Xia et al., it is shown that ginsenoside Rg3 and Rh2 can induce apoptosis and inhibit the proliferation of human leukemia cells by increasing ROS levels in mitochondria.^6^ Leukemia stem cells are responsible for the relapsing of acute leukemia, resistance to chemotherapy, and metastasis in patients. Some studies revealed that ginsenoside can target and suppress leukemia stem cells.^2^ In this review article, we want to introduce the ginsenoside derivatives and review the preclinical studies that investigated the anticancer effects of ginsenosides against leukemia cancer cells.

## Ginseng and its derivatives

Ginseng is the root or rhizome of various perennial herbaceous plants of the genus Panax, which belongs to the family Araliaceae. It has been broadly used as a valuable and popular traditional herbal medicine in East Asian countries mainly China, Korea, and Japan for thousands of years.^7^ In the past, ginseng was utilized for reinforcement energy and bodily power, enhancement of longevity, and the diminution of stress and exhaustion.^8^ There are several ginseng species of the genus Panax including Panax ginseng Meyer (Korean ginseng), Panax notoginseng (Chinese ginseng), Panax japonicum (Japanese ginseng), Panax quinquefolius (American ginseng), Panax vietnamensis (Vietnamese ginseng), Panax omeiensis (Omei ginseng), Panax zingiberensis (ginger ginseng), Panax wangianus (narrow-leaved pseudoginseng), and Panax pseudoginseng (Himalayan ginseng).^9^ Among these, Panax ginseng is one of the most common forms used to treat numerous disorders.^10^ Ginseng has several beneficial components, including ginsenosides, polysaccharides, polyacetylenes, volatile oils, vitamins, protein, amino acids, and fatty acids.^11^^,^^12^ Different types of ginseng are classified based on the growth environment, the cultivation methods (cultivated ginseng, mountain ginseng), and the preparation methods (white ginseng, red ginseng, black ginseng) such as steaming, fermentation, and drying.^13^^,^^14^

**Figure 1:**
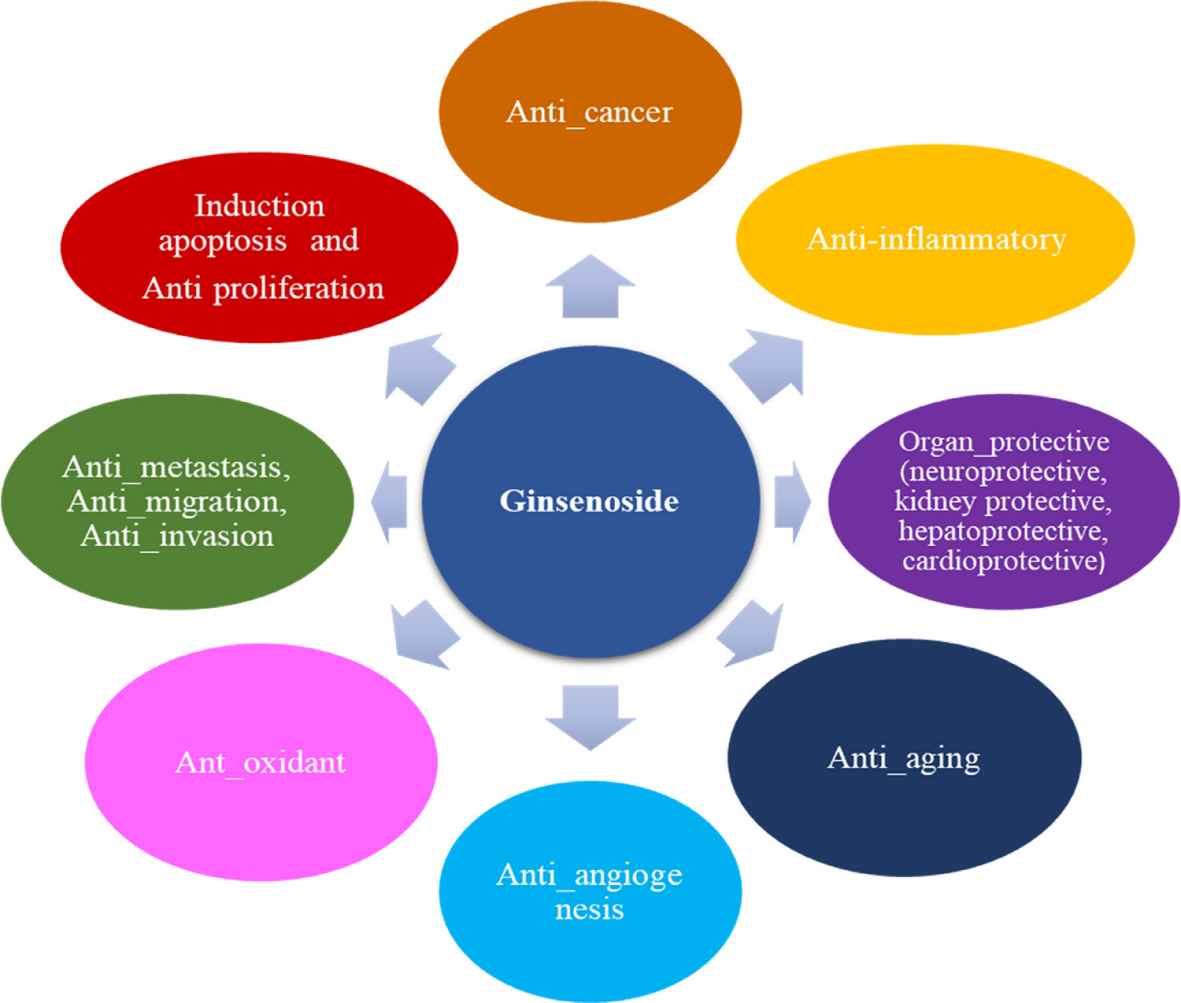
Potential biological and pharmacological activities of ginsenoside

Ginseng has numerous pharmacological and therapeutic properties such as anti-stress effects, health betterment, body invigoration, and maintaining and boosting the immune and central nervous systems. ginseng also improves memory and vision actions, alleviates heartbeats, increases intelligence, cognitive ability, and human life span, and is useful in treating common symptoms like tension, insomnia, and headache.^11^^,^^12^^,^^15^, ^16^, ^17^, ^18^, ^19^ Also, it has medicinal applications in the treatment of many diseases including the nervous system, cardiovascular diseases, metabolic diseases, and various types of cancer, and has low adverse effects.^20^ According to numerous clinical and experimental studies, Ginsenosides have a wide range of effects including anticancer properties against lung, leukemia, breast, and liver cancers, antioxidant, anti-inflammation, anti-apoptosis, anti_proliferative, and anti_metastatis. It also enhances the chemosensitivity of cancer cells, regulates metabolism, and inhibits the migration and invasion of cancer cells (Figure 1). On the other hand, ginseng has anti-aging, antivirus, antidiabetic, anti-angiogenesis, antithrombosis, and immunoregulation and organ-protective effects (neuroprotective, kidney protective, hepatoprotective, cardioprotective).^21^, ^22^, ^23^, ^24^, ^25^

Additionally, ginsenosides have many other effects in the treatment of neurological disorders including Alzheimer's disease, Parkinson's disease, epilepsy, Huntington's disease, Multiple Sclerosis, and depression.^19^^,^^26^

## Ginsenoside: Chemical Structure, Classification, and Pharmacological Effects

Ginsenosides are the main bioactive ingredient of ginseng and are the type of triterpene saponins.^16^ Until now, more than 180 types of ginsenosides have been obtained from Panax species and their pharmacological effects have been determined.^27^

**Figure 2:**
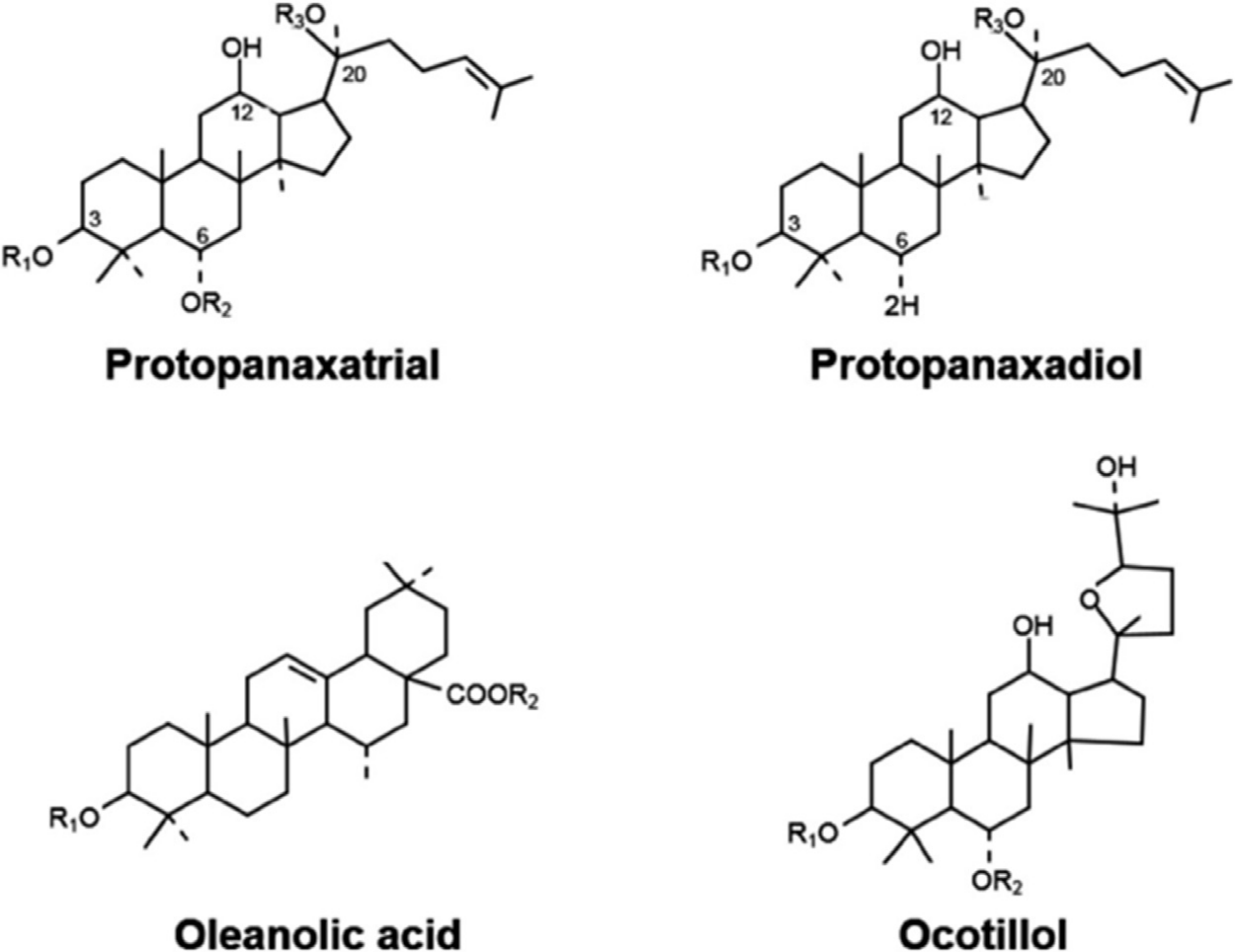
Ginsenosides Structures

**Figure 3:**
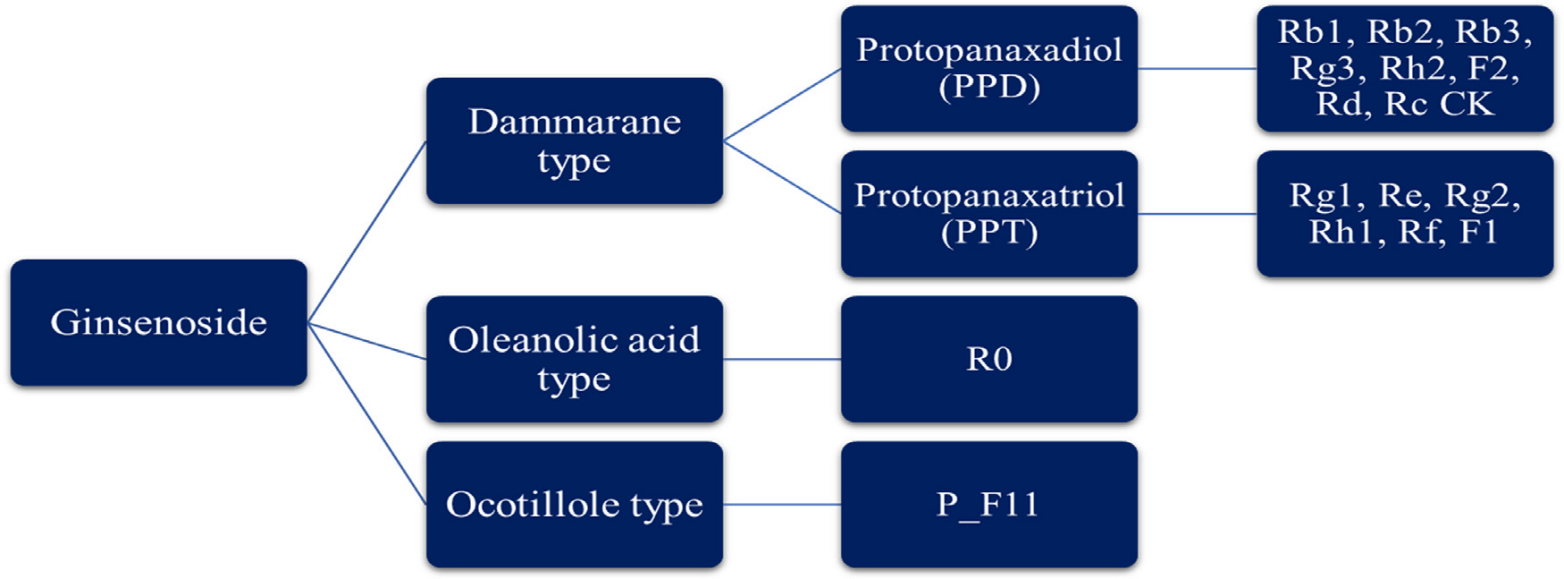
Classification of ginsenosides

The basic structure of ginsenosides is similar. All of them contain 30 carbon atoms and are composed of steroid nuclei having 17 carbon atoms arranged in four rings backbone (Aglycon moiety) (Figure 2). The type, position, and number of diverse sugar molecules (e.g., glucose, rhamnose, xylose, and arabinose) connected at C-3, C-6, and C_20 positions of the rings (Glycoside moiety) variate the ginsenosides derivatives and influences on biological as well as pharmacological effects of ginsenosides.^17^^,^^18^ Based on their structures, ginsenosides are classified into four groups: Panaxadiol group (PPD) (e.g., Ra1, Ra2, Ra3, Rb1, Rb2, Rb3, Rc, Rd, Rg3, Rh2, CK and F2), and Panaxatriol group (PPT) (e.g., Re, Rg1, Rg2, Rh1, Rf, and F1), which belong to dammarane group, Oleanolic acid group (OA) (e.g., Ro) and ocotillol group (QT) (e.g., pseudo ginsenoside_ F11).^9^^,^^11^^,^^28^ According to the positioning of hydroxyl (OH) on carbon 20 (C20), the dammarane group of ginsenosides (PPD and PPT types) has two major categories; 20(S) and 20(R) epimers include 20(S)-protopanaxadiol (PPD) and 20(S)-protopanaxatriol (PPT), 20(R)-protopanaxadiol (PPD) and 20(R)-protopanaxatriol (PPT) types (Figure 3).^29^

The PPD category is determined by the sugar molecule connection at C-3 and/or C-20 position, and the PPT category is identified by the sugar molecule connection at C-6 and/or C-20 position. The Oleanolic acid class is pentacyclic triterpenoid saponins, and made up of a pentacyclic structure with an aglycone oleanolic acid like ginsenoside Ro; while the Ocotillol class contains an epoxy ring attached to C–20, and inclusive ginsenoside R2 and the pseudo ginsenoside F11 (p-F11).^9^^,^^16^^,^^30^ PPT- and PPD-type ginsenosides are plentiful in Panax ginseng, P. quinquefolius, and P. notoginseng; and P. japonicasi, while P. vietnamensis contains OT-type ginsenosides. also, OA-type ginsenoside is abundant in other species of ginseng (P. zingiberensi, P. stipuleanatus, P. bipinnatifidus, and P. sokpayensis).^9^ So far, among ginsenosides obtained from different ginseng plants, Four PPD-type ginsenosides (Rb1, Rb2, Rc, and Rd) and two PPT-type ginsenosides (Re and Rg1) responsible for almost 90% of the total ginsenosides and are considered as the main ginsenosides.^9^^,^^16^ Due to differences in the type and amount of ginsenosides in various Panax species, there is diversity in their pharmacological activities.^9^

## Ginsenoside Rh2

The pharmacological effects of Ginsenoside Rh2 (GR2), a member of the Ginsenosides family, have been observed in various cancer types, including leukemia cells. GRh2 can cause cell cycle arrest in the G1 phase via Retinoblastoma protein phosphorylation reduction and transcription factor translocation.^31^, ^32^, ^33^ GRh2 is a potential Histone deacetylase inhibitor for leukemia treatment as it effectively induces cell cycle arrest and apoptosis, increased Histone acetyltransferase (HAT) activity and histone acetylation, and can inhibit the growth of human leukemia cells.^34^^,^^35^

Xiaoru Wang conducted a study to investigate the therapeutic potential of GRh2 in pediatric leukemia. They reported that GRh2 can suppress the Bcl-2 expression by modulating miR-21 which leads to a decrease in cell viability and increase in apoptosis. These results suggest that GRh2 could be a promising treatment option for pediatric leukemia.^36^ GRh2 was found to be capable of inducing both apoptosis and autophagy in ALL cells specifically without advert effects on normal blood cells. However, it was also found that autophagy plays a protective role in GRh2-induced apoptosis. In other words, the autophagy process helps the cells to resist GRh2-induced apoptosis. The researchers discovered that inhibiting autophagy can make ALL cells more sensitive to GRh2-induced apoptosis. In conclusion, inhibition of autophagy can sensitize ALL cells towards GRh2 and make it a more effective anti-cancer agent for ALL therapy.^37^ Also, GRh2 is considered a chemotherapeutic agent for leukemia therapy by inducing apoptosis through the mitochondrial pathway including mitochondrial depolarization, cytochrome complex release, and activation of caspase-9 and caspase-3 in acute leukemia cells.^38^

GRh2 exhibits immune regulation and anti-tumor effects in T-cell acute lymphoblastic leukemia (T-ALL) by blocking the Phosphatidylinositol 3-kinase**/** Mammalian target of rapamycin (PI3K/Akt/mTOR) signaling pathway, enhancing immunity in the spleen, promoting intestinal homeostasis, and altering the composition of gut microbiota, indicating that it may have the potential for the prevention and treatment of T-ALL.^39^ Furthermore, another study demonstrated that GRh2 can effectively inhibit the growth of T-ALL cells by inducing apoptosis and autophagy and blocking the PI3K/Akt/mTOR signaling pathway. The study showed that GRh2 could reduce T-ALL progression. These results suggest that GRh2 may have therapeutic potential for T-ALL treatment.^40^

Another study showed that GRh2 decreased NB4 cell viability in a dose- and time-dependent manner and induced apoptosis, cell cycle arrest, and caspase activation. GRh2 also upregulated Tumor necrosis factor alpha (TNF-α) expression and inhibited Akt phosphorylation, leading to Promyelocytic leukemia/Retinoic acid receptor alpha (PML/PML-RARA) degradation, PML nuclear bodies formation, and activation of the downstream p53 pathway in NB4 cells. The study concluded that GRh2 induces caspase-dependent PML-RARA degradation and apoptosis through the Akt/Bax/caspase9 and TNF-α /caspase8 pathways.^41^ GRh2 induces cell death via apoptosis and autophagy in AML and CML cells, with a stronger apoptotic effect in K562 cells. Autophagy plays a protective role against apoptosis, suggesting that combining GRh2 with autophagy inhibitors may have therapeutic potential for AML and CML.^42^ GRh2 can slow downregulation of the growth of HL-60 leukemia cells by preventing them from progressing through the G1 phase of the cell cycle and causing them to differentiate. This effect may be due to the increased expression of cyclin-dependent kinase inhibitor 1 (p21CIP1/WAF1), which inhibits the activity of cyclin-dependent kinases CDK2, CDK4, and CDK6.^43^

To summarize, it can be briefly stated that multiple studies have demonstrated the remarkable anti-cancer capabilities of GRh2 with minimal negative consequences. GRh2 can induce apoptosis and inhibit the growth of cancer cells by increasing the production of TNF-α. Additionally, it can cause G1 cell cycle arrest and differentiation in leukemia cells by elevating Transforming growth factor-beta (TGF-β) levels. Due to its various mechanisms of action such as cell cycle arrest, Histone deacetylase inhibition, up-regulation of p21CIP1/WAF1, and downregulation of CDK2, CDK4, and CDK6 activities, GRh2 is being increasingly recognized as a highly effective and hopeful treatment for leukemia with significant anti-tumor properties and limited adverse effects (Table 1).

**Table 1:** Anti-cancer effect of GRh2 on leukemia cell lines

Aim of Study	Results	References
To determine the potential of GRh2 as an HDAC inhibitor for the treatment of leukemia	-GRh2 may be a useful HDAC inhibitor for treating leukemia, as it has been shown to halt cell cycle progression, promote apoptosis, reduce cell proliferation proteins, increase HAT activity and histone acetylation, and inhibit the growth of leukemia tumors	Liu, Z.H et al. (2015)^35^
To investigate the molecular mechanisms of cell death induced by GRh2 in ALL cells	-GRh2 can effectively induce apoptosis and autophagy in ALL cells without affecting normal blood cells, and inhibiting autophagy can enhance the potency of GRh2 as an anticancer agent for ALL therapy by sensitizing ALL cells to its effects	Xia, T et al. (2016)^37^
To inspect GRh2 anti-tumor effects Related to both the immune system and the gut microbiota	-In T-ALL mice, Bacteroidetes, Verrucomicrobia, Akkermansia, Lactobacillus, and Lachnospiraceae showed positive correlations with immune parameters and gut barrier functions, while Firmicutes, Proteobacteria, Parabacteroides, and Alistipes showed negative correlations	Xia T, et al. (2020)^39^
To investigate the effects of GRh2 on the proliferation, cell cycle regulation, and differentiation of human leukemia HL-60 cells	-GRh2 inhibits the proliferation of human leukemia HL-60 cells via G1 phase cell cycle arrest and induces differentiation, possibly through up-regulation of p21CIP1/WAF1 and down-regulation of CDK2, 4, and 6	Cho, S.-H et al. (2006)^43^
To evaluate the molecular mechanisms underlying the pro-apoptotic and pro-differentiation effects of Ginsenoside GRh2 on acute myeloid leukemia cells	-GRh2 activates the expression of nuclear receptor (Nur77) and death receptor proteins Fas Cell Surface Death Receptor (Fas), Fas Ligand (FasL), Death Receptor 5 (DR5), and Tumor necrosing factor-Related Apoptosis-Inducing Ligand (TRAIL), led to the apoptosis and differentiation of AML cells	Wang, C et al. (2017)^57^
To investigate the effects of ginsenosides, such as Protopanaxadiol ginsenoside (PD), Protopanaxatriol ginsenoside (PT), and their derivatives Rh2 and Rh1, on the human leukemia cells proliferation	-GRh2 was effective in inhibiting the proliferation of human leukemia cells and inducing apoptosis and DNA fragmentation, but its effects were lower than those of PD and PT treatments	Popovich, D.G et al. (2002)^18^
To evaluate the impact of GRh2 on human acute T lymphoblastic leukemia Jurkat cells and the underlying mechanism	-GRh2 inhibited Jurkat cell proliferation in a dose-time dependent manner, induced apoptosis and cell cycle block in the G0/G1 phase, and promoted the expression of BAX and Caspase-3 while inhibiting the expression of BCL-2, Cyclin D1, and p-AKT, with PI3K inhibitor LY294002 enhancing these effects	Nie L, Peng HM. (2019)^58^
To analyze the effect of GRh2 on leukemia KG1α cells	-Rh2 is the most effective ginsenoside on the growth of KG1α cells -GRh2 had the lowest Half-maximal inhibitory concentration (IC50) compared to Rb1, Rg1 and cytarabine	You Z, et al. (2014)^59^
To investigate the effect of GRh2 on inducing apoptosis of human leukemia K562 cells	-GRh2 inhibited cell proliferation, induced apoptosis and autophagy, and activated p-p38, which could be weakened by an autophagy inhibitor	Liu XX, et al. (2017)^60^
To inspect how GRh2 can inhibit human leukemia cells and understand how it works in terms of autophagy and apoptosis	-Ginsenoside Rh₂ inhibits human leukemia cell growth in vivo through the regulation of autophagy and apoptosis via the HDAC6 and Heat shock protein 90 pathways	Liu ZH, et al. (2016)^61^
To investigate the effects of GRh2 on cell proliferation, HDAC1 and HDAC2 activity, and expression of cyclin in human erythroleukemia K562 cells	-GRh2 inhibited the proliferation of K562 cells, induced cell cycle arrest, and apoptosis, and reduced HDAC activity through downregulating cyclin D1 and activating cyclin-dependent kinase inhibitor 2A (p16INK4A) and p21	Xia J, et al. (2014)^62^

## Ginsenoside Rg3

Ginsenoside Rg3 (GRg3) is a type of ginsenoside found in high quantities. Numerous studies have shown that GRg3 has the potential to prevent inflammation, diabetes, and cardiovascular diseases.^44^, ^45^, ^46^, ^47^ GRg3 has been found to have anti-cancer effects in many types of cancer. In short, it induces apoptosis, arrests the cell cycle, inhibits the growth, proliferation, and metastasis of cancer cells, and prevents angiogenesis against cancer cells.^48^

Based on previous studies, GRg3 induces apoptosis in leukemia by downregulating Phosphatidylinositol 3-kinase (PI3K/Akt) family proteins.^49^ Also, GRg3 has been found to lower vascular endothelial growth factor (VEGF) expression in acute leukemia patients by deactivating the PI3K/Akt and extracellular signal-regulated kinase ½ (ERK1/2) pathways.^50^ In another research, GRg3 triggered programmed cell death in Jurkat cells and resulted in the formation of condensed nuclei and apoptotic bodies. It also increases the expression levels of apoptosis-associated proteins, such as caspase-3 and -9, and reduces the expression of VEGF. It can inhibit cell proliferation by inducing excessive mitochondrial reactive oxygen species (ROS), which results in a reduction in mitochondrial membrane potential.^51^

GRg3 can be potentially used as a supplementary treatment for leukemia. Multidrug resistance (MDR) poses a major obstacle to the effectiveness of current cytotoxic drugs in achieving optimal outcomes for cancer treatment. GRg3 has been shown to enhance the effects of anticancer agents in MDR cells by modulating efflux-mediated drug accumulation defects. Specifically, 20(S)-Rg3 exhibits the most potent inhibitory activity on MDR by inhibiting the drug efflux pump and increasing drug accumulation in MDR cells. Overall, GRg3 has the potential to enhance the effectiveness of chemotherapy in cancer treatment.^52^ In a separate study, the co-administration of GRg3 and doxorubicin using a co-loaded biomimetic nanosystem resulted in increased tumor immunogenicity and improved immune system response, suggesting its potential as an adjuvant treatment for AML.^53^

In summary, GRg3 has demonstrated remarkable anti-tumor effects in various studies with minimal side effects. Moreover, it improves the therapeutic results and efficacy of chemotherapy and radiotherapy by affecting multiple antioxidant, anti-inflammatory, and immune signaling pathways. Additionally, it inhibits tumor cell proliferation and neovascularization, promotes tumor cell apoptosis, and reverses tumor chemoresistance. In light of its demonstrated remarkable anti-tumor functions, minimal side effects, and multiple mechanisms of action, GRg3 is increasingly recognized as a promising and effective antitumor agent in the field of oncology (Table 2).

**Table 2:** Anti-cancer effect of GRg3 on leukemia cell lines

Aim of Study	Results	References
To explore the effect of 20-(s)-GRg3 on apoptosis in human leukemic U937 and HL-60 cells and the underlying mechanism.	-20-(S)-GRg3 induced apoptosis in both U937 and HL-60 cells in a dose-dependent manner and this effect was attributed to the activation of caspase-3 and the downregulation of Bcl-2 expression. -20-(S)-GRg3 induce the generation of reactive oxygen species.	Xiao-Min et al. (2014)^49^
Investigation of the anti-angiogenic effects of Rg3 in patients with acute leukemia.	-Rg3 inhibited the expression of HIF-1α and VEGF in BMSCs through the downregulation of Akt and ERK1/2 phosphorylation.	Dongfeng Zeng et al. (2014)^50^
Investigation of the anticancer properties of GRh2 and GRg3 in Jurkat cells.	-GRh2 and GRg3 inhibited cell growth and induced apoptosis, but GRh2 had greater cytotoxicity than GRg3. -The expression levels of apoptosis-associated proteins were significantly increased in Jurkat cells treated with GRh2 compared to GRg3.	Ting Xia et al. (2017)^51^
To determine whether an interaction between the two compounds would allow lower concentrations to be used clinically to modulate drug resistance.	-20S-Rg3 may be used as a Vp synergize or as a promising alternative to Vp in the chemosensitization of multidrug-resistant acute myeloid leukemia, with far fewer side effects.	Sung Su et al. (2014)^52^
explain the impacts of ginsenosides in non-neoplastic conditions and neoplastic conditions	-The viability of human leukemic cells is reduced and apoptosis is induced by 20-(S)-GRg3 through the increased activity of caspase-3 and caspase-9.	Ghafouri‑Fard et al. (2022)^63^
To introduce a novel biomimetic nanosystem that can co-load doxorubicin and ginsenoside and be used for chemoimmunotherapy of acute myeloid leukemia	- Rg3 was found to enhance tumor sensitivity to DOX, activate the anti-tumor immune response, and effectively combat leukemia cells in the bone marrow.	Mo Chen et al. (2022)^53^
To explore how GRg3 affects HIF-1α and VEGF levels in bone marrow stromal cells (BMSCs) in acute leukemia, and identify the underlying mechanism.	-Rg3 inhibits HIF-1α and VEGF expression in leukemia BMSCs and downregulates PI3K/Akt and MAPK pathways, suggesting an anti-angiogenesis role in acute leukemia bone marrow.	Wang Jin et al. (2010)^64^

## Ginsenoside Rg1

Another ginseng derivative that is used for the treatment of leukemia is Ginsenoside Rg1 (GRg1). Some studies indicated that Rg1 can induce senescence markers such as beta-galactosidase in leukemia cells.^2^^,^^54^^,^^55^ Yan et al. performed a study to investigate the anticancer effects of GRg1 on KG1α leukemia cells. they isolated the CD34+/CD38- leukemia stem cells from the KG1α cell line and divided them into control and GRg1 groups. Results show that Rg1 can significantly reduce the proliferation of stem cells compared to the control group. In addition, there were more cancer stem cells in the G0/G1 phase of the cell cycle and also fewer cancer stem cells in the G2/M and S phase of the cell cycle in the GRg1 group compared with the control group. Finally, they concluded that GRg1 induced the expression of beta-galactosidase as a senescence marker and suppressed the Sirtuin 1 and tuberous sclerosis complex 2 (TSC2) gene expression. The sirtuin 1 gene is responsible for cell proliferation and senescence and interaction with TSC2. It is shown that overexpression of Sirtuin 1 is related to resistance to treatment of leukemia.^2^ Induction of senescence in leukemia stem cells by GRg1 was observed in another study. It could increase the p16INK4a expression that has antitumor activity and also decrease the hTERT (Telomerase reverse transcriptase) expression.^55^

In 2014 Li et al. designed a study to investigate the anticancer activity of GRg1 against acute myeloid leukemia. They found that the proliferation of leukemia cancer cells is suppressed after treatment of cells with GRg1 in a dose and time-dependent manner. The effects of GRg1 on apoptosis-related genes are considerable. It was shown that GRg1 can induce apoptosis by upregulating the Bax and caspase 3 proteins and reducing the expression of EpoR (a key gene in oncogenic signaling pathways). It inactivates the JAK2/STAT5 signaling pathway which has important effects on cell proliferation, survival, and apoptosis. GRg1 toxicity wasn't observed in less than 100µM concentration (Table 3).^56^

**Table 3:** Anticancer effects of GRg1 on leukemia cancer cell lines

Aim of study	Results	References
ginsenoside effects on induction of apoptosis and inhibition of proliferation in HL-60 cell line	-GRh2 can induce apoptosis and inhibit the cancer cell's proliferation through up-regulation of TNF-α -GRh2 can cause cell cycle arrest in the G1 phase of replication	Huang et al. (2016)^21^
Inhibitory effects of GRg1 on the proliferation of leukemia stem cells through SIRT1/TSC2 signaling pathway	-After treatment with GRg1, leukemia cells in the G2/M and S phases of replications were reduced significantly whereas cells in the G0/G1 phase of replication were elevated -Mixed colony-forming unit and beta-galactosidase (senescence markers) were increased significantly -GRg1 can inhibit proliferation and induce senescence markers in leukemia stem cells through activation of the SIRT1 TSC2 signaling pathway	Tang et al. (2020)^2^
Induction of senescence in leukemia stem cells by GRg1 through the effect on p16INK4a and hTERT gene expression	-GRg1 can induce Senescence in leukemia stem cells by overexpression of p16INK4a and suppression of hTERT expression	Tang et al. (2021)^55^
Effects of GRg1 on K562 leukemia cell line	-After treatment with GRg1, the senescence-related proteins, and beta-galactosidase were increased. Also, GRg1 can inhibit proliferation and colony formation and cause shortened telomere length. It can also alter the morphology of cancer cells by increasing mitochondria size and lysosomes number	Liu et al. (2012)^65^
Anti-cancer activity of GRg1 on K562 leukemia cancer cells	-Induction of apoptosis through upregulation of Bax, CPAPR protein, caspase 3, and suppression of Bcl-2, and AG490 -Inhibition of the JAK2/STAT5 pathway and suppression of EpoR expression	Li et al. (2014)^56^
Discovering the effects of GRg1 on inducing senescence in K562 leukemia cells by	-Inhibition of K562 cell proliferation and cell cycle arrest in the G2/M phase -Upregulation of senescence-related genes (P16, P21, P53, and Rb)	Cai et al. (2012)^54^
Investigation of ginsenoside CK effects against acute myeloid leukemia	-Induction of apoptosis and G1 cell cycle arrest -Upregulation of TET2 gene expression -Downregulation of CSF-1, KIT, BCL2, MYC, and DNMT3A gene expression -In conclusion, CK is a safe and efficient agent against myeloid leukemia cells	Hou et al. (2022)^66^
Synergistic activity of ginsenoside CK and cytarabine on acute myeloid leukemia cells	-The cytotoxicity, DNA damage, and apoptosis induced by cytarabine are promoted by ginsenoside CK	Qi et al. (2020)^5^
Investigation of CK effects on human leukemia cells	-Induction of apoptosis in leukemia via caspase activation -Inhibition of leukemia cell viability	Cho et al. (2009)^67^
Investigation of ginsenoside Rk3 effects on extramedullary infiltration of AML	-Inhibition of extramedullary infiltration of cancer cells -Suppression of invasion, migration, and proliferation of SHI-1 leukemia cells -Increase the expression of mir-3677-5p	Ma et al. (2022)^68^
Anticancer effects of ginsenoside Rh1 against acute monocytic leukemia	-Decrease the expression of MCP1 and CCR2 in monocytes -Inhibition of invasion and migration of THP-1 leukemia cell line through suppression of MAPK pathway -Cytotoxicity of GRh2 against leukemia cells wasn't seen in 50µM concentration	Choi et al. (2011)^69^

## Other ginsenoside derivatives

Other forms of ginsenosides are also discussed in studies. Compound k (CK) is another ginsenoside derivative that has been used for the suppression of leukemia cells. CK can potentially target the cancer cells by different mechanisms. Telomerase has an important role in the pathogenesis of cancer cells and is activated in most types of cancer cells. CK inhibits the telomerase function and causes apoptosis and cell cycle arrest in the G1 phase.^70^ Another study shows that It can induce apoptosis in HL-60 leukemia cells by activation of caspase-3 and caspase-9 enzymes.^71^^,^^72^ In 2013, Chen et al. investigated the anti-leukemic activity of CK on pediatric myeloid leukemia cells. They assessed the proliferation, apoptosis, cell viability, and cell cycle of cancer cells after the application of CK. Results show the inhibitory effect of CK on the growth and proliferation of myeloid leukemia cells in a time and dose-dependent manner. CK induces apoptosis and cell cycle arrest and also suppresses the synthesis of DNA in cancer cells. They concluded that CK can be a potential natural medicine for the improvement of pediatric acute myeloid leukemia.^73^

In another study by Ma et al., they reported that ginsenoside Rk3 can inhibit the extramedullary infiltration of leukemia cells. Researchers measured the expression of mir-3677-5p and CXCR4 and investigated the correlation of them using Rk3. It was shown that overexpression of mir-3677-5p inhibits the invasion, migration, and proliferation of leukemia cells. In contrast, the patients with extramedullary infiltration of leukemia have higher expression of CXCR4. In the Rk3 group, the expression of mir-3677-5p was significantly higher than in the control group whereas the expression of CXCR4 was decreased compared to the control group after treatment with Rk3.^68^ Ginsenoside Rh1 is another ginsenoside derivative with anti-inflammatory and antiallergic properties. Choi et al. found that Rh1 can inhibit the migration and invasion of THP-1 cells and reduce the expression of Monocyte chemoattractant protein 1(MCP-1) and CCR2 (chemokine receptor). MCP-1 is a chemokine that is increased in untreated patients with AML.^69^^,^^74^

The combination of ginsenoside derivatives with chemotherapeutic medications is also investigated for the treatment of leukemia. For example, Qi et al. reported the anticancer effects of ginsenoside CK combined with cytarabine for the treatment of acute myeloid leukemia cells. It was shown that CK and cytarabine synergistically inhibit the proliferation of cancer cells, increase cytotoxicity, induce apoptosis, and also cause DNA damage in leukemia cells.^5^ In another investigation, Chen et al. used GRg3 with doxorubicin for the treatment of AML. They loaded these drugs into a platelet membrane-based nano-system and investigated their efficacy in the suppression of leukemia cells. They reported that the sensitivity of leukemia cells to doxorubicin increases when it is used in combination with GRg3. In addition, it was shown that a combination of GRg3 and doxorubicin can potentially activate T cells against leukemia cells. GRg3 had a synergistic effect on the immunogenic death of cancer cells when used with doxorubicin.^75^ On the other hand, some studies reported that the application of GRg1 along with doxorubicin decreases the side effects of chemotherapy such as doxorubicin-induced cardiotoxicity.^76^ A meta-analysis that analyzed the results of 18 clinical trials showed that GRg3 improves chemotherapy-induced cytopenia and recommended GRg3 for myelosuppression patients.^77^ Besides doxorubicin, it was shown that GRg3 increases the sensitivity of leukemia cells to the vincristine.^78^

**Figure 4:**
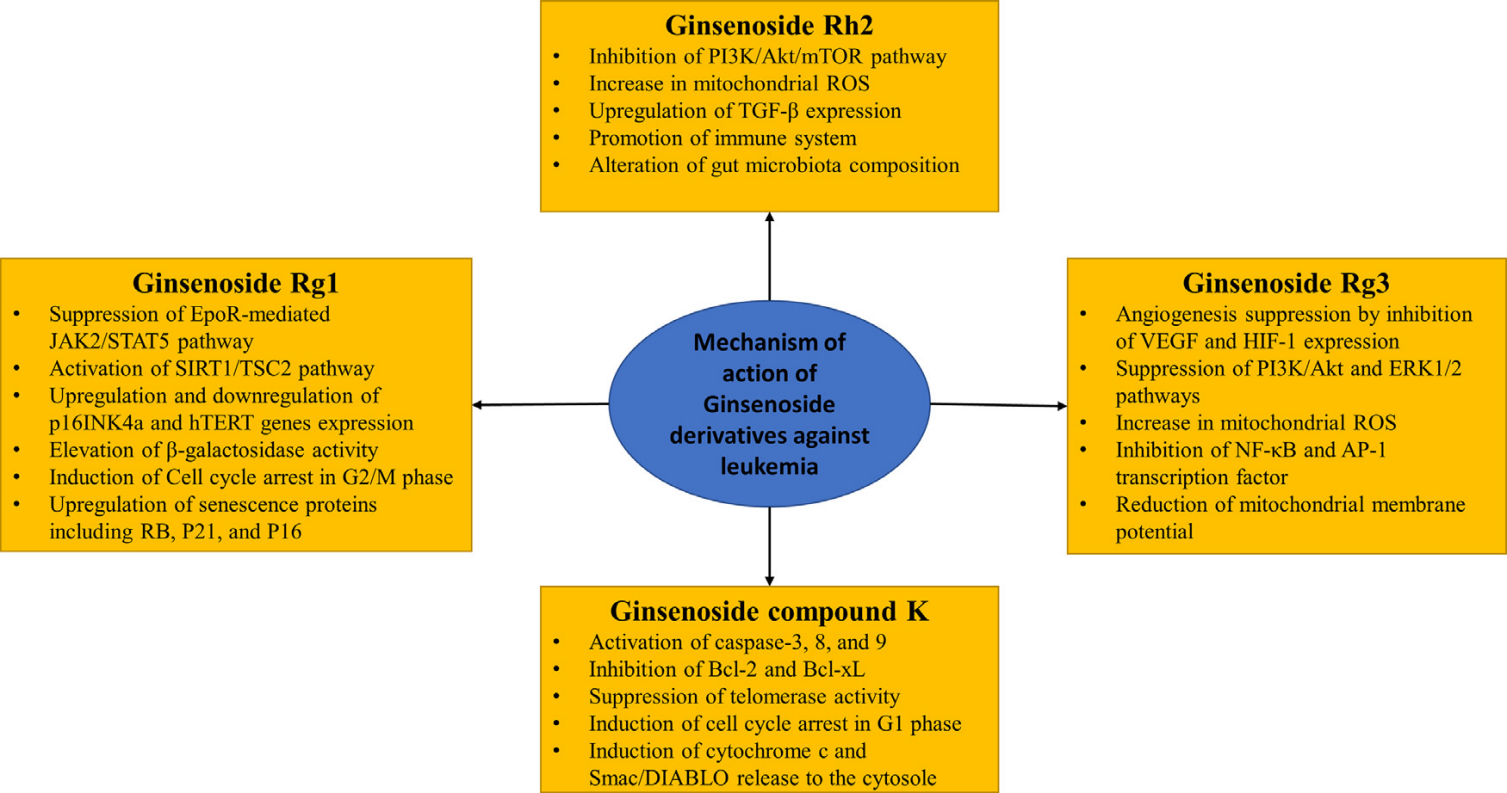
Mechanism of action of ginsenoside derivatives

In conclusion, using agents that potentiate the anticancer activity of chemotherapeutic drugs, especially in chemotherapy resistance-cancers, and decrease the adverse effects of chemotherapy are beneficial for cancer patients. Further research is needed to illustrate the efficacy of ginsenoside derivatives for the promotion of chemotherapeutic medications efficacy and preventing their side effects (Figure 4).

## Conclusion

In this study, we reviewed the anticancer potential of ginsenosides against leukemia cancer cells. Ginsenosides induce apoptosis and cell cycle arrest, suppress the proliferation of cancer cells, induce senescence markers, and suppress the leukemia cancer cells via different signaling pathways including the JAK2/STAT5 signaling pathway. However, the results of preclinical studies are promising, more studies especially clinical trial studies are required to investigate the safety and efficacy of ginsenosides on leukemia patients.

## Declaration of competing interest

There isn't any competing interest in this research.

## References

[1] Creutzig U, van den Heuvel-Eibrink MM, Gibson B (2012). Diagnosis and management of acute myeloid leukemia in children and adolescents: recommendations from an international expert panel. Blood.

[2] Tang YL, Zhang CG, Liu H (2020). Ginsenoside Rg1 Inhibits Cell Proliferation and Induces Markers of Cell Senescence in CD34+CD38- Leukemia Stem Cells Derived from KG1α Acute Myeloid Leukemia Cells by Activating the Sirtuin 1 (SIRT1)/Tuberous Sclerosis Complex 2 (TSC2) Signaling Pathway. Med Sci Monit.

[3] Tang CPS, McMullen J, Tam C (2018). Cardiac side effects of bruton tyrosine kinase (BTK) inhibitors. Leukemia & lymphoma.

[4] Thol F, Ganser A (2020). Treatment of Relapsed Acute Myeloid Leukemia. Curr Treat Options Oncol.

[5] Qi W, Yan X, Xu X (2020). The effects of cytarabine combined with ginsenoside compound K synergistically induce DNA damage in acute myeloid leukemia cells. Biomed Pharmacother.

[6] Xia T, Wang YN, Zhou CX (2017). Ginsenoside Rh2 and Rg3 inhibit cell proliferation and induce apoptosis by increasing mitochondrial reactive oxygen species in human leukemia Jurkat cells. Molecular medicine reports.

[7] Miao L, Yang Y, Li Z, Fang Z, Zhang Y, Han C-c (2022). Ginsenoside Rb2: A review of pharmacokinetics and pharmacological effects. Journal of Ginseng Research.

[8] Tam DNH, Truong DH, Nguyen TTH (2018). Ginsenoside Rh1: a systematic review of its pharmacological properties. Planta Medica.

[9] Piao XM, Huo Y, Kang JP (2020). Diversity of ginsenoside profiles produced by various processing technologies. Molecules.

[10] Surh YJ, Na HK, Lee JY, Keum YS (2001). Molecular mechanisms underlying anti-tumor promoting activities of heat-processed Panax ginseng CA Meyer. Journal of Korean medical science.

[11] Kim JH, Yi Y-S, Kim M-Y, Cho JY (2017). Role of ginsenosides, the main active components of Panax ginseng, in inflammatory responses and diseases. Journal of Ginseng Research.

[12] Liu J, Wang Y, Yu Z (2022). Functional mechanism of ginsenoside compound K on tumor growth and metastasis. Integrative cancer therapies.

[13] Lee MR, Yun BS, In OH, Sung CK (2011). Comparative study of Korean white, red, and black ginseng extract on cholinesterase inhibitory activity and cholinergic function. Journal of Ginseng research.

[14] Xu X-f, Cheng X-l, Lin Q-h (2016). Identification of mountain-cultivated ginseng and cultivated ginseng using UPLC/oa-TOF MSE with a multivariate statistical sample-profiling strategy. Journal of Ginseng Research.

[15] Bai L, Gao J, Wei F, Zhao J, Wang D, Wei J (2018). Therapeutic potential of ginsenosides as an adjuvant treatment for diabetes. Frontiers in pharmacology.

[16] Feng H, Xue M, Deng H, Cheng S, Hu Y, Zhou C (2022). Ginsenoside and its therapeutic potential for cognitive impairment. Biomolecules.

[17] Leung KW, Wong AS-T (2010). Pharmacology of ginsenosides: a literature review. Chinese medicine.

[18] Popovich DG, Kitts DD (2002). Structure–function relationship exists for ginsenosides in reducing cell proliferation and inducing apoptosis in the human leukemia (THP-1) cell line. Archives of Biochemistry and Biophysics.

[19] Zheng M, Xin Y, Li Y (2018). Ginsenosides: a potential neuroprotective agent. BioMed research international.

[20] Xu J, Pan Y, Liu Y (2021). A review of anti-tumour effects of ginsenoside in gastrointestinal cancer. Journal of Pharmacy and Pharmacology.

[21] Huang J, Peng K, Wang L (2016). Ginsenoside Rh2 inhibits proliferation and induces apoptosis in human leukemia cells via TNF-α signaling pathway. Acta Biochimica et Biophysica Sinica.

[22] Kang Z, Zhonga Y, Wu T, Huang J, Zhao H, Liu D (2021). Ginsenoside from ginseng: a promising treatment for inflammatory bowel disease. Pharmacological Reports.

[23] Sun M, Ye Y, Xiao L, Duan X, Zhang Y, Zhang H (2017). Anticancer effects of ginsenoside Rg3. International journal of molecular medicine.

[24] Wang Z, Ding M, Lin Z, He C, Zhao Y (2019). Esterified Derivatives of Panaxadiol and Their Inhibitory Effect on HL-60, THP-1, and PC-3 Cell Lines. Chemistry & Biodiversity.

[25] Zha W, Sun Y, Gong W, Li L, Kim W, Li H (2022). Ginseng and ginsenosides: Therapeutic potential for sarcopenia. Biomedicine & Pharmacotherapy.

[26] Khan S, Tosun A, Kim YS (2015). Ginsenosides as food supplements and their potential role in immunological and neurodegenerative disorders. *Bioactive nutraceuticals and dietary supplements in neurological and brain disease*: Elsevier.

[27] Yang J-L, Hu Z-F, Zhang T-T, Gu A-D, Gong T, Zhu P (2018). Progress on the studies of the key enzymes of ginsenoside biosynthesis. Molecules.

[28] Nasimi Doost Azgomi R (2023). The role of ginseng derivatives against chemotherapy-induced cardiotoxicity; a systematic review of non-clinical studies. Frontiers in Cardiovascular Medicine.

[29] Nakhjavani M, Smith E, Townsend AR, Price TJ, Hardingham JE. (2020). Anti-angiogenic properties of ginsenoside Rg3. Molecules.

[30] Fuzzati N (2004). Analysis methods of ginsenosides. Journal of Chromatography B.

[31] Chen Y, Liu ZH, Xia J (2016). 20(S)-ginsenoside Rh2 inhibits the proliferation and induces the apoptosis of KG-1a cells through the Wnt/β-catenin signaling pathway. Oncology reports.

[32] Chung KS, Cho SH, Shin JS (2013). Ginsenoside Rh2 induces cell cycle arrest and differentiation in human leukemia cells by upregulating TGF-β expression. Carcinogenesis.

[33] Huang J, Peng K, Wang L (2016). Ginsenoside Rh2 inhibits proliferation and induces apoptosis in human leukemia cells via TNF-α signaling pathway. Acta biochimica et biophysica Sinica.

[34] Kitts DD, Popovich DG, Hu C (2007). Characterizing the mechanism for ginsenoside-induced cytotoxicity in cultured leukemia (THP-1) cells. Canadian journal of physiology and pharmacology.

[35] Liu ZH, Li J, Xia J (2015). Ginsenoside 20(s)-Rh2 as potent natural histone deacetylase inhibitors suppressing the growth of human leukemia cells. Chemico-biological interactions.

[36] Wang X, Wang Y (2015). Ginsenoside Rh2 Mitigates Pediatric Leukemia Through Suppression of Bcl-2 in Leukemia Cells. Cellular physiology and biochemistry: international journal of experimental cellular physiology, biochemistry, and pharmacology.

[37] Xia T, Wang J, Wang Y (2016). Inhibition of autophagy potentiates anticancer property of 20(S)-ginsenoside Rh2 by promoting mitochondria-dependent apoptosis in human acute lymphoblastic leukaemia cells. Oncotarget.

[38] Xia T, Wang JC, Xu W (2014). 20S-Ginsenoside Rh2 induces apoptosis in human Leukaemia Reh cells through mitochondrial signaling pathways. Biological & pharmaceutical bulletin.

[39] Xia T, Zhang B, Li Y (2020). New insight into 20(S)-ginsenoside Rh2 against T-cell acute lymphoblastic leukemia associated with the gut microbiota and the immune system. European journal of medicinal chemistry.

[40] Xia T, Zhang J, Zhou C (2020). 20(S)-Ginsenoside Rh2 displays efficacy against T-cell acute lymphoblastic leukemia through the PI3K/Akt/mTOR signal pathway. Journal of ginseng research.

[41] Zhu S, Liu X, Xue M (2021). 20(S)-ginsenoside Rh2 induces caspase-dependent promyelocytic leukemia-retinoic acid receptor A degradation in NB4 cells via Akt/Bax/caspase9 and TNF-α/caspase8 signaling cascades. Journal of ginseng research.

[42] Zhuang J, Yin J, Xu C, Mu Y, Lv S (2018). 20(S)-Ginsenoside Rh2 Induce the Apoptosis and Autophagy in U937 and K562 Cells. Nutrients.

[43] Cho S-H, Kim D-H, Lee K-T (2006). Proceedings of the Ginseng society Conference.

[44] Jeong D, Irfan M, Kim S-D (2017). Ginsenoside Rg3-enriched red ginseng extract inhibits platelet activation and in vivo thrombus formation. Journal of Ginseng Research.

[45] Park Y-J, Cho M, Choi G, Na H, Chung Y (2020). A critical regulation of Th17 cell responses and autoimmune neuro-inflammation by ginsenoside Rg3. Biomolecules.

[46] Wang H, Wu W, Wang G (2019). Protective effect of ginsenoside Rg3 on lung injury in diabetic rats. Journal of cellular biochemistry.

[47] Zhou T, Sun L, Yang S (2020). 20(S)-Ginsenoside Rg3 protects kidney from diabetic kidney disease via renal inflammation depression in diabetic rats. Journal of diabetes research.

[48] Liu Z, Liu T, Li W, Li J, Wang C, Zhang K (2021). Insights into the antitumor mechanism of ginsenosides Rg3. Mol Biol Rep.

[49] Qiu X-M, Bai X, Jiang H-F, He P, Wang J-H (2014). 20-(s)-ginsenoside Rg3 induces apoptotic cell death in human leukemic U937 and HL-60 cells through PI3K/Akt pathways. Anti-Cancer Drugs.

[50] Zeng D, Wang J, Kong P, Chang C, Li J, Li J (2014). Ginsenoside Rg3 inhibits HIF-1α and VEGF expression in patient with acute leukemia via inhibiting the activation of PI3K/Akt and ERK1/2 pathways. Int J Clin Exp Pathol.

[51] Xia T, Wang YN, Zhou CX (2017). Ginsenoside Rh2 and Rg3 inhibit cell proliferation and induce apoptosis by increasing mitochondrial reactive oxygen species in human leukemia Jurkat cells. Mol Med Rep.

[52] Kim SS, Seong S, Kim SY (2014). Synergistic effect of ginsenoside Rg3 with verapamil on the modulation of multidrug resistance in human acute myeloid leukemia cells. Oncology Letters.

[53] Chen M, Qiao Y, Cao J, Ta L, Ci T, Ke X (2022). Biomimetic doxorubicin/ginsenoside co-loading nanosystem for chemoimmunotherapy of acute myeloid leukemia. J Nanobiotechnology.

[54] Cai S, Zhou Y, Liu J, Liu D, Jiang R, Wang Y (2012). [Experimental study on human leukemia cell line K562 senescence induced by ginsenoside Rg1]. Zhongguo Zhong Yao Za Zhi.

[55] Tang YL, Wang XB, Zhou Y, Wang YP, Ding JC (2021). Ginsenoside Rg1 induces senescence of leukemic stem cells by upregulating p16INK4a and downregulating hTERT expression. Adv Clin Exp Med.

[56] Li J, Wei Q, Zuo GW (2014). Ginsenoside Rg1 induces apoptosis through inhibition of the EpoR-mediated JAK2/STAT5 signalling pathway in the TF-1/Epo human leukemia cell line. Asian Pac J Cancer Prev.

[57] Wang C, He H, Dou G (2017). Ginsenoside 20(S)-Rh2 induces apoptosis and differentiation of acute myeloid leukemia cells: role of orphan nuclear receptor Nur77. Journal of agricultural and food chemistry.

[58] Nie L, Peng HM (2019). [Apoptosis-Inducing Effect of Ginsenoside Rh2 on Human Acute T Lymphoblastic Leukemia Jurkat Cells and Its Mechanism]. Zhongguo shi yan xue ye xue za zhi.

[59] You Z, Chen D, Wei Q (2014). [Ginsenoside Rh2 inhibits proliferation and promotes apoptosis of leukemia KG1-α cells]. Xi Bao Yu Fen Zi Mian Yi Xue Za Zhi.

[60] Liu XX, Xia J, Tang JF, Zhou MH, Chen DL, Liu ZH (2017). [Ginsenoside Rh₂ induces apoptosis and autophagy of K562 cells by activating p38]. Zhongguo Zhong yao za zhi = Zhongguo zhongyao zazhi = China journal of Chinese materia medica.

[61] Liu ZH, Chen DL, Jiang R (2016). [Ginsenoside Rh₂-induced inhibition of histone deacetylase 6 promotes K562 cells autophagy and apoptosis in vivo]. Zhongguo Zhong yao za zhi = Zhongguo zhongyao zazhi = China journal of Chinese materia medica.

[62] Xia J, Chen D, Zuo G (2014). [Regulatory effect of ginsenoside Rh2 on HDAC1/2 activity and cyclin in human erythroleukemia K562 cells]. Xi bao yu fen zi mian yi xue za zhi = Chinese journal of cellular and molecular immunology.

[63] Ghafouri-Fard S, Balaei N, Shoorei H (2022). The effects of Ginsenosides on PI3K/AKT signaling pathway. Mol Biol Rep.

[64] Wang J, Kong P, Xu W, Zeng D (2010). Ginsenosisde Rg3 inhibits HIF-1α and VEGF expressions in acute leukemia bone marrow stromal cells. Acta Academiae Medicinae Militaris Tertiae.

[65] Liu J, Cai SZ, Zhou Y (2012). Senescence as a consequence of ginsenoside rg1 response on k562 human leukemia cell line. Asian Pac J Cancer Prev.

[66] Hou Y, Meng X, Sun K (2022). Anti-cancer effects of ginsenoside CK on acute myeloid leukemia in vitro and in vivo. Heliyon.

[67] Cho SH, Chung KS, Choi JH, Kim DH, Lee KT (2009). Compound K, a metabolite of ginseng saponin, induces apoptosis via caspase-8-dependent pathway in HL-60 human leukemia cells. BMC Cancer.

[68] Ma S, Huang Q, Hu Q (2022). Ginsenoside Rk3 Inhibits the Extramedullary Infiltration of Acute Monocytic Leukemia Cell via miR-3677-5p/CXCL12 Axis. Evidence-Based Complementary and Alternative Medicine.

[69] Choi Y-J, Yoon J-H, Cha S-W, Lee S-G (2011). Ginsenoside Rh1 inhibits the invasion and migration of THP-1 acute monocytic leukemia cells via inactivation of the MAPK signaling pathway. Fitoterapia.

[70] Kang KA, Lee KH, Chae S (2006). Inhibition of telomerase activity in U937 human monocytic leukemia cells by compound K, a ginseng saponin metabolite. Biotechnology and Bioprocess Engineering.

[71] Cho S-H, Chung K-S, Choi J-H, Kim D-H, Lee K-T (2009). Compound K, a metabolite of ginseng saponin, induces apoptosis via caspase-8-dependent pathway in HL-60 human leukemia cells. BMC cancer.

[72] Kang KA, Kim YW, Kim SU (2005). G 1 phase arrest of the cell cycle by a ginseng metabolite, compound K, in U937 human monocytic leukamia cells. Archives of pharmacal research.

[73] Chen Y, Xu Y, Zhu Y, Li X (2013). Anti-cancer effects of ginsenoside compound k on pediatric acute myeloid leukemia cells. Cancer cell international.

[74] Mazur G, Wrobel T, Butrym A, Kapelko-Słowik K, Poreba R, Kuliczkowski K (2007). Increased monocyte chemoattractant protein 1 (MCP-1/CCL-2) serum level in acute myeloid leukemia. Neoplasma.

[75] Chen M, Qiao Y, Cao J, Ta L, Ci T, Ke X (2022). Biomimetic doxorubicin/ginsenoside co-loading nanosystem for chemoimmunotherapy of acute myeloid leukemia. Journal of Nanobiotechnology.

[76] Xu Z-M, Li C-B, Liu Q-L, Li P, Yang H (2018). Ginsenoside Rg1 prevents doxorubicin-induced cardiotoxicity through the inhibition of autophagy and endoplasmic reticulum stress in mice. International journal of molecular sciences.

[77] Pan L, Zhang T, Cao H, Sun H, Liu G (2020). Ginsenoside Rg3 for chemotherapy-induced myelosuppression: A meta-analysis and systematic review. Frontiers in Pharmacology.

[78] Hou YJ, Hao F, Liu XD, Yuan ZH, Li Y (2013). Rg3 Monomer as a Chemotherapy Drugs Sensitizer to Acute Leukemia Cells. Advanced Materials Research.

